# Novel monitoring technique to minimise the risk for patients participating in pilot studies of investigational compounds

**DOI:** 10.1186/1758-2652-13-S4-P228

**Published:** 2010-11-08

**Authors:** R Cuffe, M Ait-Khaled, S Hughes, S Min, G Nichols, D Thomas, M Underwood, JM Yeo

**Affiliations:** 1 GlaxoSmithKline, Stockley Park West, Middlesex, UK; 2 ViiV Healthcare, Middlesex, UK; 3 GlaxoSmithKline, Research Triangle Park, USA

## Background

S/GSK1349572 is a new integrase inhibitor (INI) with in-vitro activity against INI-resistant HIV. Before dosing a large number of subjects in pivotal clinical trials, in-vitro findings were validated in a pilot clinical study (ING112961). At the outset of the study, the degree of susceptibility at which the drug no longer provides benefit was unknown. Planned enrollment for this study was 30 raltegravir (RAL)-resistant patients, incorporating a broad range of in-vitro susceptibility to S/GSK1349572. The clinical team developed novel stopping guidelines to minimise risk to study participants.

## Methods

"Unacceptable" response rates were elicited for key numbers of recruited subjects (e.g. 5, 20, 30). These rates were translated statistically into a level of evidence for unacceptable efficacy, measured as a likelihood ratio (LR). The LR defined a stopping boundary. This boundary was defined for every assessment of response and was tested after each patient's results were observed.

## Results

LR stopping guidelines are shown in Table 1 and compared with the rule "stop for >70% failures". A fixed 70% failure rate threshold does not allow for accumulating evidence. LR thresholds stop for lower rates of failure as more data are collected.

**Table 1:** Number of observed treatment failures that would stop the study

Number of patients observed	4	10	16	20	26	30
70% failure stopping threshold	3	7	12	14	19	21

LR stopping threshold	4 (100%)	5 (50%)	7 (44%)	8 (40%)	9 (35%)	10 (33%)

At Day 11, 21/27 (78%) of subjects with resistance to raltegravir and elvitegravir showed a virologic response. However, five of the six failures were from the nine subjects enrolled with decreased susceptibility to GSK1349572. The failure rate in this group (5/9) met the definition of "strong evidence of non-response" according to the LR thresholds and so enrollment into this group was halted early.

## Conclusions

Monitoring response in a patient enables best treatment for that individual. In trials of new treatments, determining the best option for the next patient requires interpretation of accumulating data in population-level monitoring. This challenging task requires a combination of clinical insight and formal quantification of evidence. This case study shows that likelihood ratio thresholds define "strong evidence" of non-response and are suitable for constant monitoring. This approach enables additional safety checks for the best treatment for subjects when a new treatment has accumulated only limited data.

